# Metabolic Profiling of Fermented Products of the Ethanolic Extract of *Acanthopanax sessiliflorus* Fruit and Evaluation of Its Immune Enhancement Effect in RAW 264.7 Macrophages and BV2 Microglia

**DOI:** 10.3390/antiox14040397

**Published:** 2025-03-27

**Authors:** Kwan-Woo Kim, Bo-Ram Choi, Woo-Cheol Shin, Jin-Kyu Jang, Young-Seob Lee, Dahye Yoon, Dae Young Lee

**Affiliations:** 1 Department of Herbal Crop Research, National Institute of Horticultural and Herbal Science, Rural Development Administration, Eumseong 27709, Republic of Korea; 2 BK21 FOUR KNU Creative BioResearch Group, School of Life Sciences, Kyungpook National University, Daegu 41566, Republic of Korea

**Keywords:** *Acanthopanax sessiliflorus* fruit, fermentation, prebiotics, fermented product, immune enhancement effect, macrophage, microglia

## Abstract

In this study, we sought to evaluate the potential availability of 30% ethanol extract of *Acanthopanax sessiliflorus* fruit (ASE) as a prebiotic and compare the immune enhancement effect of ASE and its fermented products, which were fermented with three probiotic bacteria, namely, *Lactobacillus plantarum* (ASE-LPF), *Streptococcus thermophilus* (ASE-STF), and *Lactobacillus helveticus* (ASE-LHF). RAW264.7 and BV2 cells were treated with various concentrations of ASE and its fermented products. The level of nitric oxide was evaluated using a Griess reagent, and the levels of inflammatory cytokines were determined through an enzyme-linked immunosorbent assay. Western blot analysis was employed to determine the expression of various proteins related to immune responses. Our results show that fermentation with ASE significantly improved the probiotic growth of *S. thermophilus* and *L. helveticus*. Compared with ASE, treatment with only ASE-LHF increased the level of nitric oxide. Compared with ASE, treatment with ASE-LHF augmented the expression of inducible nitric oxide synthase, cyclooxygenase-2, and the production of inflammatory cytokines. It was confirmed that these enhancement effects were due to the activation of the nuclear factor kappa B and extracellular signal-regulated kinase mitogen-activated protein kinase signaling pathways. Additionally, secondary metabolite profiling of ASE and its fermented products was performed using UPLC-QTOF/MS to identify ASE’s promising compounds. Through metabolomic analysis, 23 metabolites showing significant differences between ASE and its fermented products were compared. Therefore, this study demonstrates the possibility of ASE-LHF as a potential material for immune-enhancing agents.

## 1. Introduction

An immune response refers to a reaction that occurs in order for an organism to defend itself against external or foreign materials, including viruses, bacteria, parasites, and fungi, which could cause serious problems to the health of the host organisms [1]. It is divided into innate immunity (natural immunity) from birth and acquired immunity, which is obtained through infection or vaccination. In particular, the innate immunity system is regulated by various immune cells, such as neutrophils, macrophages, and monocytes, and soluble factors, such as cytokines and complements [2]. Among them, macrophages are the most dominant and widely distributed immune cells in the body and play a critical role in innate and adaptive immune responses for protecting the host against pathogens and external materials [3]. When the body is stimulated by pathological foreign materials, macrophages are activated, and they secrete various inflammatory mediators, including nitric oxide (NO), prostaglandin E2 (PGE_2_), and pro-inflammatory cytokines such as interleukin (IL)-1β, IL-6, tumor necrosis factor (TNF)-α, interferon, and chemokines [4,5]. These inflammatory mediators can feed back to regulate or activate the immune cells, phagocytizing and neutralizing them to restore the health of cells and tissues [6]. Therefore, the activation of macrophages initiates an immune response and induces antigen processing and presentation to promote adaptive immunity [7]. Microglial cells are a type of neuroglia located throughout the brain and spinal cord, which account for 10~15% of all cells found within the brain [8]. They are considered resident macrophages in the central nervous system (CNS) and act as the first and main form of active immune defense in the CNS [9]. Similar to macrophages, microglial cells perform a variety of tasks within the CNS related to both immune response and maintaining homeostasis, including scavenging and phagocytosis, which are the main roles of microglia, presentation of antigen, and promotion of repair [10,11,12].

The *Acanthopanax* species is a deciduous shrub belonging to the Araliaceae family, is widely distributed across Korea, Japan, and China, and has been used as a traditional medicinal ingredient for the treatment of various diseases, including diabetes, tumors, rheumatoid arthritis, hypertension, and liver injury [13,14]. The leaves, roots, root bark, stems, and fruits of this species contain various ingredients such as lignan, coumarin, diterpene, triterpene, phenolic compounds, and saponin, which are attracting attention as valuable medicinal plant resources [15]. More than 18 *Acanthopanax* species inhabiting East Asia are known to grow in Korea, among which is *Acanthopanax sessiliflorus* [16]. This plant contains various substances such as lignan, triterpenoidal saponin, triterpene, coumarin, flavonoids, and phenylpropanoids [17,18,19,20].

Probiotics are live microorganisms that provide health benefits to the host by stimulating native gut microbiota [21]. They basically inhibit harmful bacteria in the intestine and multiply beneficial bacteria, and show health functionality such as anti-diabetes, anti-obesity, anti-inflammatory, anti-cancer, and anti-allergic [22,23]. The most consumed probiotics are known as strains of two species, *Bifidobacteria* and *Lactobacillus*, and according to the Health Food Code, several species, including *Lactobacillus*, *Lactiplantibacillus*, and *Streptococcus*, can be used as raw materials for probiotic products. Prebiotics are compounds in food that induce the growth or activity of beneficial microorganisms such as bacteria and fungi [24]. They can be obtained naturally from various plants such as onion, asparagus, garlic, chicory, oat, and wheat and promote metabolic activities in the colon by stimulating bacterial growth [21]. Research has shown that using growth media enriched with extracts from polyphenol-rich plants like Willow gentian, St. John’s wort, winter savory, and yarrow can enhance the growth of probiotic strains and improve the intestinal microbial environment [25]. However, there is limited research on inoculating various natural products, such as medicinal crops, with probiotic strains and culturing them. Furthermore, there are no documented cases of cultivating beneficial strains using extracts of medicinal crops as a standalone growth medium. Accordingly, through this study, we tried to determine whether medicinal crops including *A. sesslilflorus* fruits can play a role as prebiotics to promote the growth of probiotic strains. In addition, the interest in research to improve immunity with natural products has been highlighted recently, and in particular, immunoenhancing agents derived from natural products are expected to strengthen immune responses or restore reduced immunity [26]. Therefore, we aimed to investigate the immune-enhancing properties of the ethanolic extract of *A. sesslilflorus* fruits (ASE) and their fermented byproducts to explore the potential of using *A. sesslilflorus* fruits as an immune-boosting agent.

## 2. Materials and Methods

### 2.1. Chemicals and Reagents

RPMI1640, fetal bovine serum (FBS), and other tissue culture reagents were purchased from Gibco BRL Co. (Grand Island, NY, USA). Other reagents for cell culture, including lipopolysaccharide (LPS) and 3-(4,5-dimethylthiazol-2-yl)-2,5-diphenyltetrazolium bromide (MTT), were obtained from Sigma-Aldrich (St. Louis, MO, USA). Primary antibodies, including anti-inducible nitric oxide synthase (iNOS), anti-cyclooxygenase-2 (COX-2), anti-inhibitor kappa B (IκB)-α, anti-p-IκB-α, anti-phospho-extracellular signal-regulated kinase (ERK), anti-ERK, anti-phospho-c-Jun N-terminal kinase (JNK), anti-JNK, anti-phospho-p38, and anti-p38, were purchased from Cell Signaling Technology (Danvers, MA, USA), and anti-β-actin was obtained from Santa Cruz Biotechnology (Dallas, TX, USA). Secondary antibodies such as anti-rabbit and anti-mouse were purchased from Merck Millipore (Darmstadt, Germany).

### 2.2. Preparation of ASE and Its Fermented Products and Evaluation of Probiotic Growth Using Fermentation with ASE

The preparation of ASE was conducted based on a previous report [27]. *A. sessiliflorus* fruits were harvested in Jeongseon, Republic of Korea, and extracted under reflux for 6 h using 30% aqueous fermented ethanol at 70 °C for 6 h and extracted again for 3 h under the same conditions. After filtering using a 5 μm filter, the extract was concentrated under reduced pressure to obtain 10–20 brix materials. Concentrated extract was pasteurized at 80–90 °C for 1 h and then freeze-dried under reduced pressure (−30 °C, 100 mTorr) for 24 h. The extract was diluted with 0.1% peptone water with a final concentration of 100 µg/mL and then inoculated with three probiotic strains, including *Lactobacillus plantarum*, *Streptococcus thermophilus*, and *Lactobacillus helveticus*, with 1 × 10^3^ colony forming units (CFUs)/mL. The probiotic strains including *L. plantarum*, *S. thermophilus*, and *L. helveticus* were obtained from the Korean Agricultural Culture Collection (KACC) established in the Rural Development Administration (RDA). The extract and bacterial mixture were fermented for 24, 48, and 72 h at 37 °C in a humidified atmosphere containing 5% CO_2_. After fermentation was completed, 1 mL of the mixture was diluted in 0.1% peptone water, then transferred to a De Man, Rogosa, and Sharpe agar (MRS agar) medium, and cultured for an additional 24 h. After counting the number of probiotic strains transferred to the MRS agar, the final concentration was determined based on the dilution ratio. The remaining mixture was filtered through a filter paper to remove probiotic strains, concentrated, and then freeze-dried under reduced pressure conditions for application to cell models.

### 2.3. Cell Culture

The RAW264.7 macrophage cell line was purchased from the American Type Culture Collection (ATCC) and BV2 microglial cells were provided by Professor Youn-Chul Kim of Wonkwang University (Iksan, Republic of Korea). RAW264.7 macrophages and BV2 microglial cells were cultured in 100 mm diameter dishes at a density of 5 × 10^6^ cells per dish or 5 × 10^5^ cells/mL. The culture medium consisted of RPMI1640 supplemented with 10% (*v*/*v*) heat-inactivated fetal bovine serum (FBS), penicillin G (100 units/mL), streptomycin (100 µg/mL), and L-glutamine (2 mM). The cells were maintained at 37 °C in a humidified environment with 5% CO_2_.

### 2.4. MTT Assay for Cell Viability

RAW264.7 macrophages and BV2 microglial cells were treated with ASE and its fermented products at concentrations of 12.5–200 μg/mL for 24 h. Cell viability was determined by adding 2.5 mg/mL of MTT so that the final concentration could be 0.5 mg/mL with additional incubation for 3 h. The resulting formazan was dissolved in dimethyl sulfoxide, and the optical density was measured at a 540 nm wavelength. The optical density of the formazan solution from the control group (untreated group) was considered to indicate 100% viability. The formula for relative viability is [28]:

$$\mathrm{Relative}\;\mathrm{viability}\;(\%)=\frac{Sample\;absorbance}{Control\;absorbance}\;\times \;100$$

### 2.5. Determination of Nitrite (NO Production)

To measure the nitrite concentration in the culture medium, which serves as an indicator of NO production, a Griess reaction was employed. A 100 µL aliquot from each culture medium was mixed with an equal volume of Griess reagent, which consists of 0.1% (*w*/*v*) N-(1-naphthyl)-ethylenediamine and 1% (*w*/*v*) sulfanilamide in 5% (*v*/*v*) phosphoric acid. The mixture was incubated for 10 min at room temperature. The absorbance of the resulting product was then measured spectrophotometrically at 540 nm using an ELISA plate reader. The nitrite concentration in the samples was calculated based on a standard curve prepared with sodium nitrite in phosphate-buffered saline (PBS). The formula of the sodium nitrite standard curve used in this investigation is as follows:

Absorbance (540 nm wavelength) = 0.0579189 × (Concentration) + 0.00606

### 2.6. Assays for IL-6 and TNF-α

The culture media were collected to determine the levels of IL-6 and TNF-α present in each sample using profit ELISA kits (R&D systems Inc., Minneapolis, MN, USA). Three independent experiments were performed according to the manufacturer’s instructions.

### 2.7. Western Blot Analysis

RAW264.7 and BV2 cells were collected and centrifuged at 16,000 rpm for 15 min to form a pellet. The cells were then washed with PBS and lysed using a 20 mM Tris-HCl buffer (pH 7.4) that contained protease inhibitors, including 0.1 mM phenylmethylsulfonylfluoride (PMSF), 5 mg/mL aprotinin, 5 mg/mL pepstatin A, and 1 mg/mL chymostatin. The protein concentration was determined using a Bradford assay. Subsequently, 30 µg of protein from each sample was separated by 7.5% and 12% sodium dodecyl sulfate/polyacrylamide gel electrophoresis (SDS-PAGE) and transferred onto a nitrocellulose membrane (Bio-Rad, Hercules, CA, USA). The membrane was blocked with 5% skimmed milk and sequentially incubated with primary antibodies and horseradish peroxidase-conjugated secondary antibodies. The proteins were visualized using Hybond enhanced chemiluminescence (ECL) detection (GE Healthcare, Amersham, UK). The data presented are representative of three independent experiments and were quantified through densitometric analysis.

### 2.8. Preparation of Cytosolic and Nuclear Extract

Nuclear and cytosolic fractions of the cells were obtained using the NE-PER Nuclear and Cytoplasmic Extraction Reagents (Thermo Fisher Scientific, Waltham, WA, USA). Each extract was subsequently lysed in accordance with the manufacturer’s instructions.

### 2.9. Statistical Analysis

The data are presented as the mean ± standard deviation (SD) from at least three independent experiments. For comparisons involving three or more groups, one-way analysis of variance (ANOVA) was employed, followed by a Dunnett’s test for multi-to-one comparisons against the control group. Statistical analyses were conducted using GraphPad Prism software, version 3.03 (GraphPad Software Inc., San Diego, CA, USA).

### 2.10. UPLC-QTOF/MS-Based Secondary Metabolite Profiling of ASE and Its Fermented Products

Ultra-high-performance liquid chromatography quadrupole time-of-flight mass spectrometry (UPLC-QTOF/MS) was adopted for profiling secondary metatolites of ASE and its fermented products. UPLC was performed using a Waters ACQUITY H-Class UPLC system (Waters Corp., Milford, MA, USA) with an Acquity BEH C18 column (2.1 mm × 100 mm; 1.7 µm) for separation. The temperature of the column and auto sampler was maintained at 40 and 10 °C, respectively. The mobile phases consisted of solvent A (water with 0.1% formic acid(*v*/*v*)) and solvent B (acetonitrile with 0.1% formic acid(*v*/*v*)). The flow rate was set to 450 µL/min, and the injection volume was 2 µL. The elution conditions were as follows: 0–4 min, B 10–30%; 4–15 min, B 30–60%; 15–16 min, B 60–100%; 16–18 min, B 100–10%. MS analysis was performed using a Waters Xevo G2-S QTOF MS (Waters Corp.) in the negative ion mode. The MS data were acquired in the MSE mode, which alternates high- and low-energy scans. During data acquisition, leucine encephalin (*m*/*z* 554.262) was infused to confirm accurate mass measurement. The operating MS parameters were optimized for the analysis. The source temperature and desolvation temperature were maintained at 120 °C and 550 °C, respectively. The cone gas flow ratewas 30 L/h and the gas flow rate was 800 L/h. The capillary and cone voltage were set to 3 kV and 40 V, respectively. All MSE data were acquired through MassLynx 4.1 software (Waters Corp.) and then processed using UNIFI software (Ver.18, Waters Corp.).

## 3. Results

### 3.1. Growth of Probiotic Strains Using Fermentation with ASE

**Figure 1:**
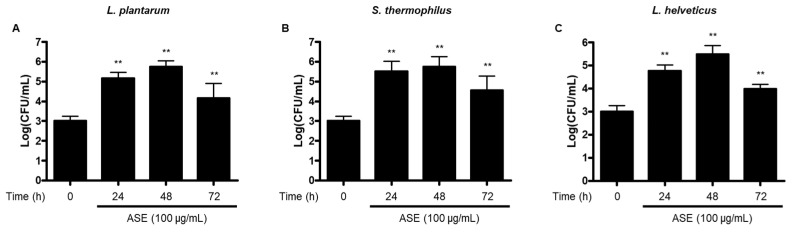
Bacterial growth of *L. plantarum* (**A**), *S. thermophilus* (**B**), and *L. helveticus* (**C**) using fermentation with ASE. ** *p* < 0.01 compared to 0 h group.

We showed that the effective ingredients contained in ASE were changed in the fermentation process using probiotics, resulting in enhanced immune activity. Therefore, we evaluated whether ASE affects the growth of the probiotic strains *L. plantarum*, *S. thermophilus*, and *L. helveticus*. In 100 μg/mL concentration conditions, the number of colonies of *L. plantarum*, *S. thermophilus*, and *L. helveticus* gradually increased by 48 h after fermentation compared to before fermentation. However, after 72 h of fermentation, the concentration of probiotic strains decreased compared to that after 24 or 48 h (Figure 1). Based on these results, we confirmed the availability of *A. sessiliflorus* fruits to improve intestinal health as a prebiotic material by promoting the growth of beneficial bacteria. Furthermore, based on the change in the growth pattern of probiotic strains after 72 h, we hypothesized that the byproducts would exhibit different activity from that of ASE. Therefore, to examine the immune enhancement activity of fermentation products produced after optimal fermentation conditions, we decided to use fermented products after 72 h of fermentation.

### 3.2. Effect of ASE and Its Fermented Products on Viability of RAW264.7 Macrophages and BV2 Microglial Cells

**Figure 2:**
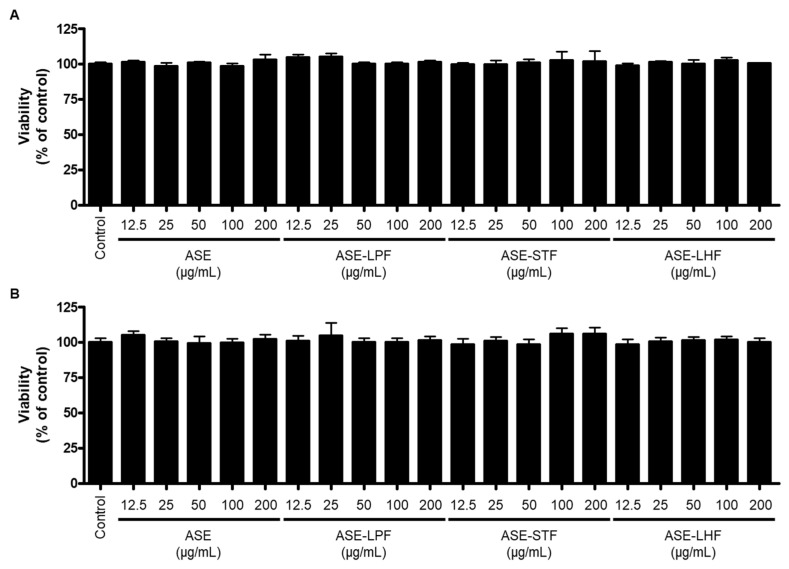
Effect of ASE and its fermented products on cell viability of RAW264.7 macrophages (**A**) and BV2 microglial cells (**B**). Cells were treated with ASE and its fermented products at the indicated concentrations for 24 h, and cell viability was determined with MTT assay.

To determine the cytotoxicity of ASE and its fermented products for this investigation, we conducted an MTT assay to evaluate the viability of RAW264.7 macrophages and BV2 microglial cells treated with ASE and its fermented products (ASE-LPF, ASE-STF, and ASE-LHF). As shown in Figure 2, the non-cytotoxic concentration range was determined to be 0.0–200 µg/mL of ASE and its three fermented products.

### 3.3. Effect of ASE and Fermented Products on Production of NO in RAW264.7 Macrophages and BV2 Microglial Cells

**Figure 3:**
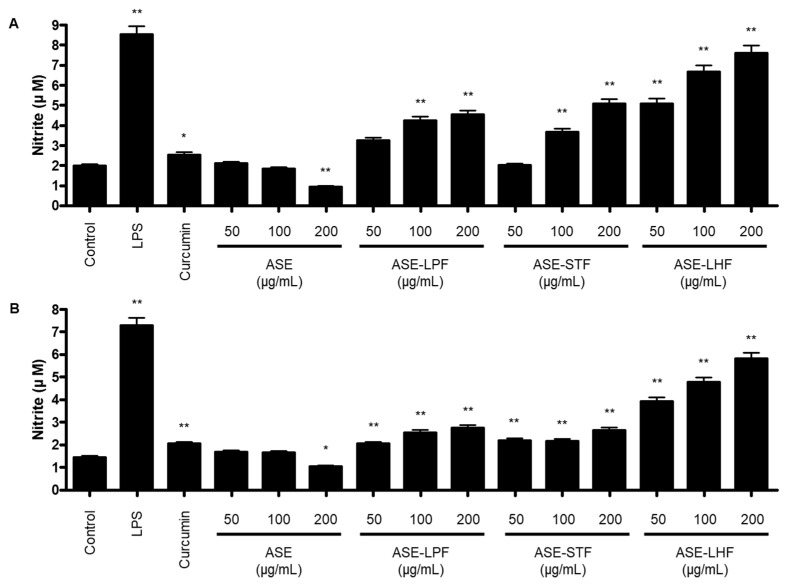
Effect of ASE and its fermented products on production of nitrite in RAW264.7 macrophages (**A**) and BV2 microglial cells (**B**). Cells were treated with LPS, curcumin, ASE, or its fermented products for 24 h. Nitrite levels were determined using the Griess reaction. Values shown are mean values ± SD of three independent experiments. * *p* < 0.05, and ** *p* < 0.01 in comparison with the control group.

Firstly, we investigated the release of NO following treatment with ASE and its fermented products in RAW264.7 macrophages and BV2 microglial cells using a Griess reagent. Cells were treated with LPS, curcumin, ASE, and fermented products of ASE incubated for 72 h. In RAW264.7 macrophages, the production of NO was not caused by the treatment with ASE. However, it was increased by fermented products with three probiotic strains compared with the control group. Among them, the concentration of NO incubated with the *L. helveticus* group was the highest of all the groups, and at a concentration of 200 µg/mL, it was 3.84 times higher than that of the control group (Figure 3A). In BV2 cells, the fermented products of ASE increased the level of NO production compared to the ASE-treated group. Similarly with the results in RAW264.7 macrophages, the concentration of NO incubated with the *L. helveticus* group was significantly increased (Figure 3B). These results suggest that the fermentation process of the ASE using probiotic strains shows an immune enhancement effect by increasing the production of NO, which ASE did not induce. Based on these results, we investigated the molecular mechanism of the immune-enhancing effect of ASE-LHF, which significantly increased NO production in both RAW264.7 and BV2 cells. Although this experiment showed a significantly better effect at a concentration of 200 μg/mL, the highest concentration was set at 100 μg/mL for subsequent experiments. This decision was made with consideration for future in vivo studies, clinical trials, and food development using the ASE and its fermented byproducts, where safety and efficacy need to be balanced.

### 3.4. Effect of ASE and ASE-LHF on Expression of iNOS and COX-2 Proteins in RAW264.7 Macrophages and BV2 Microglial Cells

**Figure 4:**
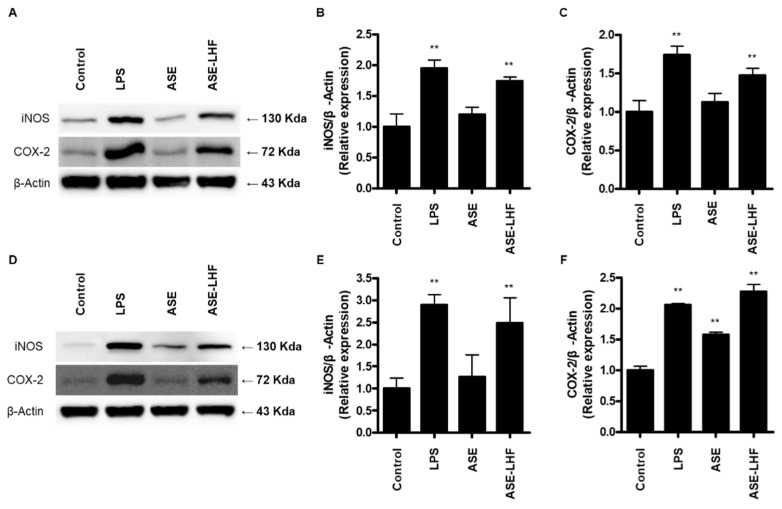
Effect of ASE and ASE-LHF on expression of iNOS and COX-2 proteins in RAW264.7 macrophages (**A**–**C**) and BV2 microglial cells (**D**–**F**). Lysates were prepared from cells treated with the indicated concentrations of LPS, ASE, or ASE-LHF for 24 h. The protein expression of iNOS and COX-2 was determined with Western blot analysis. Representative blots from three independent experiments are shown. ** *p* < 0.01 in comparison with the control group.

According to the previous results, we compared the effect of ASE and ASE-LHF on the expression of iNOS and COX-2 proteins. Cells were treated with ASE (100 μg/mL) and ASE-LHF (100 μg/mL) for 24 h. The treatment concentration of ASE and ASE-LHF, 100 μg/mL, was set in preparation for the in vivo study and clinical trial to be conducted later. As shown in Figure 4, treatment with ASE-LHF significantly enhanced the expression of iNOS and COX-2 proteins in both RAW264.7 and BV2 cells, but ASE did not affect these responses.

### 3.5. Effect of ASE and ASE-LHF on Production of Inflammatory Cytokines in RAW264.7 Macrophages and BV2 Microglial Cells

**Figure 5:**
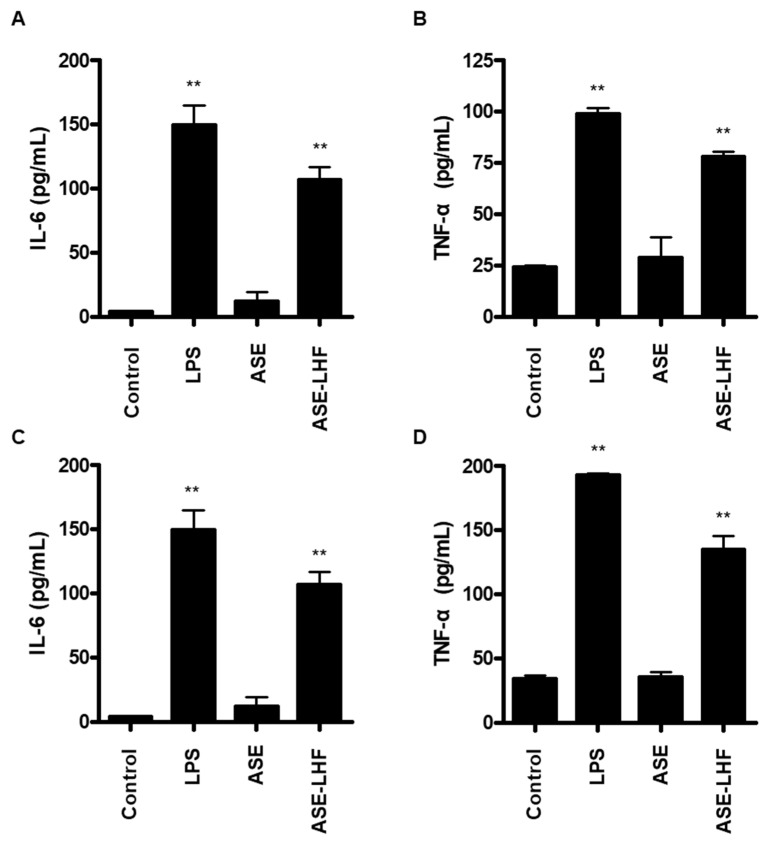
Effect of ASE and ASE-LHF on production of inflammatory cytokines in RAW264.7 macrophages (**A**) and BV2 microglial cells (**B**). Cells were treated with LPS, ASE, or ASE-LHF for 24 h. The level of IL-6 (**C**) and TNF-α (**D**) was determined and quantified with ELISA. Values shown are mean values ± SD of three independent experiments. ** *p* < 0.01 in comparison with the control group.

Additionally, we compared the effect of ASE and ASE-LHF on the production of inflammatory cytokines, including IL-6 and TNF-α. As with the previous results, ASE-LHF induced the production of IL-6 and TNF-α in both RAW264.7 and BV2 cells. However, ASE failed to induce the secretion of both IL-6 and TNF-α (Figure 5).

### 3.6. Effect of ASE-LHF on Activation of NF-κB Signaling Pathway in RAW264.7 Macrophages and BV2 Microglial Cells

**Figure 6:**
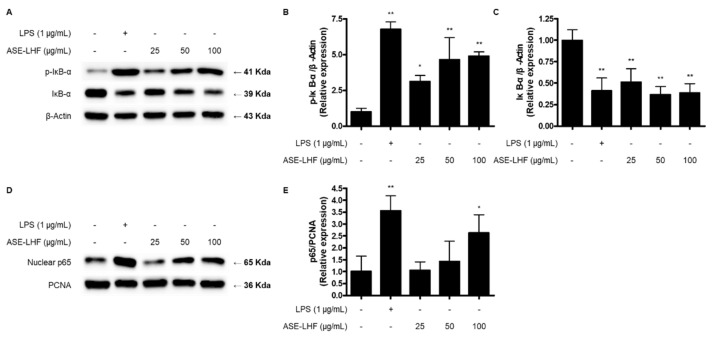

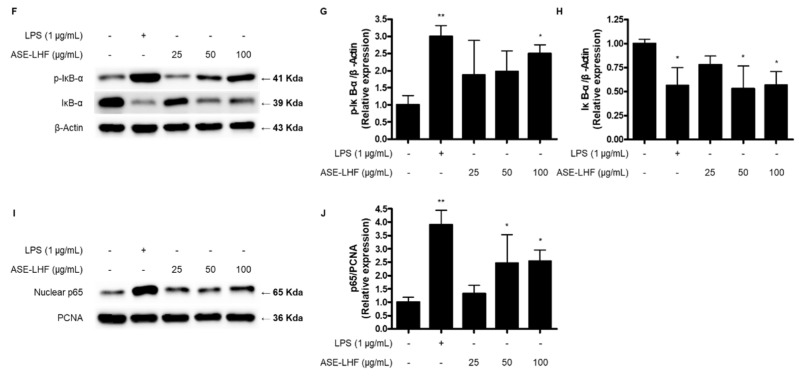
Effect of ASE-LHF on activation of NF-κB signaling pathway in RAW264.7 (**A**–**E**) and BV2 (**F**–**J**) cells. Cells were treated with LPS or ASE-LHF for 1 h, and nuclear and cytosolic extracts were isolated and the levels of p-IκB-α and IκB-α in the cytosolic fraction and p65 in the nuclear fraction were determined with Western blot analysis. β-Actin and PCNA were used as internal controls. Representative blots from three independent experiments are shown. * *p* < 0.05, and ** *p* < 0.01 in comparison with the control group.

We investigated how ASE-LHF induced the production and expression of factors related to immune responses. At first, the effect of ASE-LHF on the activation of the NF-κB signaling pathway was examined. RAW264.7 and BV2 cells were treated with the indicated concentration of LPS (1 μg/mL) or ASE-LHF for 1 h. Treatment with ASE-LHF induced the phosphorylation and degradation of IκB-α and the nuclear accumulation of the p65 subunit (Figure 6).

### 3.7. Effect of ASE-LHF on Activation of MAPK Signaling Pathway in RAW264.7 Macrophages and BV2 Microglial Cells

**Figure 7:**
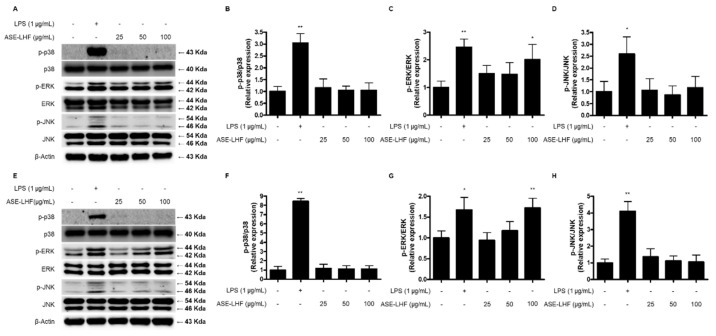
Effect of ASE-LHF on activation of MAPK signaling pathway in RAW264.7 (**A**–**D**) and BV2 (**E**–**H**) cells. Lysates were prepared from cells treated with the indicated concentrations of LPS and ASE-LHF for 1 h. Protein expression of proteins related to MAPK was determined with Western blot analysis. Representative blots from three independent experiments are shown. * *p* < 0.05, and ** *p* < 0.01 in comparison with the control group.

We also investigated the effect of ASE-LHF on the activation of the MAPK signaling pathway. RAW264.7 and BV2 cells were treated with the indicated concentration of ASE-LHF or LPS (1 µg/mL) for 1 h. As shown in Figure 7, treatment with ASE-LHF increased the phosphorylation of ERK MAPK in a dose-dependent manner in both RAW264.7 and BV2 cells. However, it had no effect on p38 and JNK MAPKs.

### 3.8. Secondary Metabolite Profiling of ASE and Its Fermented Products

**Figure 8:**
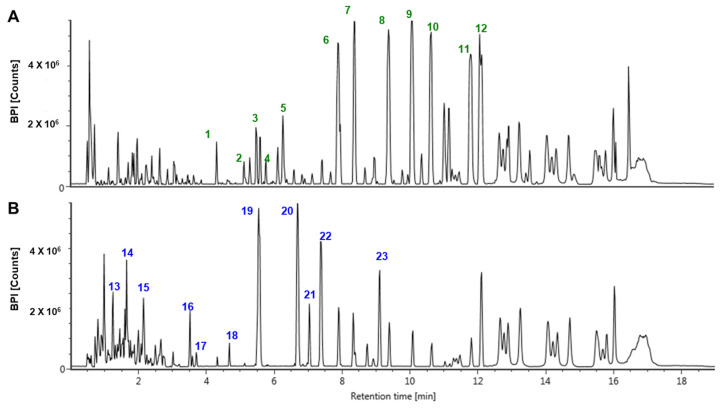
UPLC-QTOF/MS chromatograms of (**A**) ASE and (**B**) its fermented products with annotation of 23 identified metabolites.

UPLC-QTOF/MS analysis was performed to compare the secondary metabolites of ASE and its fermented products (Figure 8). Chromatograms of ASE and its fermentation products differed significantly. The acquired secondary metabolites were identified by confirming the MS values and ionization patterns with references in previous studies [28,29,30,31,32,33,34,35,36,37,38]. Information on the 23 metabolites identified in ASE and its fermented product is presented in Table 1. Compared with the fermented product of ASE, 12 characteristic metabolites of ASE were identified, including acanthosessilioside H, acanthopanaxoside E, and laciniatoside V. Most of the 12 metabolites were identified as terpenoids and they are known to have various pharmacological effects, including antitumor, anti-inflammatory, and antibacterial activity [39]. Also, in the previous study, acanthosessilioside H showed neuroinflammatory effects by significantly inhibiting the increase in nitrite concentration in both LPS-treated BV2 and RAW264.7 cells [33]. In addition, 11 characteristic metabolites including caffeoylquinic acid hexoside and 3-O-caffeoyl-4-O-pcoumaroylquinic acid were identified from the fermented product of ASE. Most of the 11 metabolites were identified as organic acids or their glycosides. Caffeoylquinic acid, an ester of caffeic acid and quinic acid, is a biological active metabolite. Caffeoylquinic acid is known to be widely distributed in medicinal plants and has been reported to have various activities including antioxidant [40], antibacterial [41], and neuroprotective activity [42]. Caffeoylquinic acid is an electron-rich compound containing phenolic hydroxyl groups, which may act as a direct antioxidant or as a regulator to oxidative stress [43]. As a result of the analysis, the metabolite with the highest relative percentages in ASE was silphioside G. The RT and m/z of the metabolites that were relatively abundant in fermented ASE were 3.52 and 819.39, respectively. The relative percentage of this metabolite was found to be 7.8%. The relative contents of these metabolites showed significant differences before and after fermentation.

## 4. Discussion

This study indicates that the ethanolic extract of *A. sessiliflorus* fruits (ASE) has potential as a prebiotic material by promoting the growth of probiotic strains, and the fermented product of ASE incubated with *L. helveticus* (ASE-LHF) exhibits immune enhancement activity in RAW264.7 macrophages and BV2 microglial cells. Treatment with ASE-LHF increased the production of NO according to the induction of iNOS and COX-2 proteins. In addition, it augmented the levels of inflammatory cytokines, including IL-6 and TNF-α, in both RAW264.7 and BV2 cells. These enhancement effects were regulated by the activation of the NF-κB and ERK MAPK signaling pathways. In addition, as a result of secondary metabolite analysis using UPLC-QTOF/MS, it was confirmed that phenolic compounds increased when ASE was fermented with *L. helveticus*, which is predicted to be associated with enhanced immune activity.

Prebiotics were first defined as nondigestible food ingredients that beneficially affect the host by selectively stimulating the growth and/or activity of one or a limited number of bacteria in the colon to improve host health [44]. In 2008, a panel of experts from the International Scientific Association for Probiotics and Prebiotics (ISAPP) updated the definition of dietary prebiotics from a wider perspective as a material that is selectively fermented and benefits the health of the host by changing the composition and activity of intestinal microorganisms [45]. Recently, the concept of prebiotics was expanded to include a substrate that is selectively utilized by host microorganisms, conferring a health benefit [46]. Prebiotics are defined as nondigestible carbohydrates that meet the following three criteria: (1) resistant to gastric acidity and hydrolysis by mammalian enzymes and gastrointestinal absorption; (2) fermented by intestinal microflora; and (3) selectively stimulate the growth and/or activity of intestinal bacteria associated with health and wellbeing [47,48]. Prebiotics are metabolized into short-chain fatty acids (SCFAs) by intestinal microorganisms such as lactic acid, butyric acid, and propionic acid, and have a small molecular weight, enabling systemic circulation through the blood stream and affecting gastrointestinal tracts and other organs and tissues [49]. In this investigation, we evaluated whether ASE has the potential to be used as a prebiotic to enhance the growth of probiotic strains through fermentation with ASE and three probiotic strains, namely, *L. plantarum*, *S. thermophilus*, and *L. helveticus*. Our results show that ASE promoted the growth of three probiotic strains (Figure 1), indicating the availability of ASE as a prebiotic substance. The concentration of all probiotic strains was higher after 48 h than after 72 h of fermentation. This seems to be a result of the probiotic strains consuming all the growth promotion-inducing components (prebiotic candidate substances) contained in ASE. Since the purpose of this study was to compare the immune enhancement effects before and after fermentation was completed, the byproducts after 72 h of fermentation were used in further experiments.

NO has been demonstrated to be a crucial and versatile intercellular messenger in the regulation of vascular tone, neurotransmission, and tissue restoration [50]. Generation of NO is a feature of genuine immune system cells, including macrophages and microglia, and other cells involved in immune reactions, including endothelial cells, mesangial cells, and Schwann cells [51]. It is involved in innate immunity as a toxic agent toward infectious organisms, including viruses, bacteria, and fungi [52], as a regulator for apoptosis of tumor cells [53], and is involved in the function of host immune cells [54], thereby regulating specific immunity. Therefore, we determined whether ASE and byproducts of fermentation with ASE (ASE-LPF, ASE-STF, and ASE-LHF) induce the production of NO as an important marker of the immune enhancement effect. As a result, it was confirmed that the production of NO was significantly increased in both RAW264.7 macrophages and BV2 microglial cells through the treatment of fermented products compared to ASE, and in particular, the responses of the byproducts with *L. helveticus* (ASE-LHF) were the highest (Figure 2). Based on these results, we selected ASE-LHF as the subject substance for the molecular mechanism study of immune enhancement activity and conducted further experiments.

NO is synthesized from L-arginine by the enzymatic activity of nitric oxide synthase (NOS). NOS has three different isoforms: neuronal NOS (nNOS), inducible NOS (iNOS), and endothelial NOS (eNOS). Unlike nNOS and eNOS, which are calcium-dependent and are continuously secreted at low concentrations, iNOS is calcium-independent and can be expressed by a variety of cells after induction with cytokines or other stimuli [55,56]. Various substances exhibiting immune-enhancing effects increased the expression of iNOS proteins in macrophages [57,58]. The expression of COX-2 protein is responsible for the production of PGE_2_, which is another important marker in the inflammatory process [59]. PGE_2_ belongs to the family of eicosanoids that have important roles in inflammatory and physiological functions, and they are small-molecule derivatives of arachidonic acid through the enzymatic activity of COX [60]. The COX proteins contain two active sites: a cyclooxygenase site, where arachidonic acid is converted into hydroperoxyl endoperoxide prostaglandin G2 (PGG_2_); a peroxidase site, which is responsible for the reduction of PGG2 to prostaglandin H2 (PGH_2_). PGH_2_ is converted to PGE_2_ due to the enzymatic action of PGE synthase [61]. It is well known that the COX protein consists of two isoforms: constitutive COX-1 and inducible COX-2. The latter is expressed in most normal mammalian tissues in response to various extracellular and intracellular physiological stimuli [62]. According to our results, treatment with ASE-LHF markedly increased the expression levels of iNOS and COX-2 proteins in both RAW264.7 and BV2 cells (Figure 4), but treatment with ASE did not affect their expression.

Cytokines are small, secreted proteins produced by every cell, and it is well known that the secretion of pro-inflammatory cytokines from immune cells is an important process in host defense [63]. Cytokines are low-molecular-weight signaling molecules that are as important to life as hormones and neurotransmitters [64]. They are secreted from various immune cells and have an important role in cell-to-cell communication, inflammatory response amplification, and immune response regulation [63,65]. Pro-inflammatory cytokines are produced predominantly by macrophages and monocytes and are involved in the growth, cell activation, differentiation, and homing of the immune cells to the sites of infection with the aim of controlling and eradicating intracellular pathogens [66]. IL-6 and TNF-α are one of the major pro-inflammatory cytokines. Our results confirm that treatment with ASE-LHF induced the production of IL-6 and TNF-α in both RAW264.7 and BV2 cells (Figure 5) compared with treatment with ASE. These results further confirm that the fermented product of ASE activates macrophages and microglial cells to enhance immunity. In summary, ASE-LHF can be considered an effective activator of macrophages and microglial cells.

NF-κB is one of the major transcription factors related to immune responses and plays a significant role in the induction of gene expression of pro-inflammatory mediators [67]. Under basal conditions, NF-κB is present in the cytoplasm with its inhibitory protein IκB-α. The activation of NF-κB is initiated by the phosphorylation and degradation of IκB-α, following the dissociation of p65 and p50 dimers, which are the main constituents of NF-κB from IκB-α, and the translocation into the nucleus [68]. In the nucleus, NF-κB dimers bind to the κB binding site of target genes and induce the transcription of pro-inflammatory mediators [69]. Our results show that the fermented product of *A. sessiliflorus* fruit activates RAW264.7 macrophages and BV2 microglial cells by inducing the phosphorylation and degradation of IκB-α (Figure 6A,B), and the translocation into the nucleus of the p65 subunit (Figure 6C,D), demonstrating that the ASE-LHF acts as a potent macrophage and microglia activator, boosting the NF-κB signaling pathway.

MAPKs are a family of serine/threonine protein kinases, and they are composed of three well-defined subfamilies, namely, p38, ERK, and JNK. MAPK regulates several cellular processes, including proliferation, stress response, apoptosis, and immune defense [70]. In addition, the activation of the MAPK signaling pathway is considered one of the important regulators in activated macrophages [4,71]. It has been reported that activation of the MAPK pathway in macrophages and microglial cells increases the secretion of inflammatory mediators and improves immune responses [71,72]. In our study, the phosphorylation of ERK MAPK increased dose-dependently with treatment with ASE-LHF in both RAW264.7 and BV2 cells (Figure 7). These results confirm that ASE-LHF induced the activation of macrophages and microglial cells by boosting the ERK MAPK signaling pathway.

To compare the metabolic differences between ASE and fermented ASE, UPLC-QTOF/MS analysis was performed. According to a previous study [73], which described the differential metabolite before and after fermentation of *Acanthopanax senticosus*, caffeic acid 4-O-glucuronide, a glycoside containing sugars, was found to significantly increase after fermentation. This seems to be similar to the increase in sugar-containing metabolites such as 1-O-p-hydroxybenzoyl-β-D-apiofuranosyl-(1→6)-β-D-glucopyranoside and [R-(E)]-1-[8-(β-D-glucopyranosyloxy)-2,6-dimethyl-2-octenoate] β-D-glucopyranose, in our study. In addition, phenolic compounds such as 3-O-caffeoyl-4-O-pcoumaroylquinic acid and 5-O-caffeoyl-3-O-pcoumaroylquinic acid, which were found to increase after fermentation compared to before fermentation, the result of enzymes decomposing the bound phenolic acids produced during the fermentation process. Moreover, during the fermentation process, phenols and bioactive compounds attached to the cell wall and cell components before fermentation were liberated [74]. The increase in phenolic compounds is related to the improved antioxidant activity [75], which may explain why ASE showed higher NO detection in the fermented state in our study.

The experiments conducted in this investigation were performed by referring to the “Guideline for functionality evaluation of Health/Functional Food provided by the Ministry of Food and Drug Safety”. In this study, we conducted experiments related to “the activation of macrophages” among biomarkers presented in the guidelines. In addition, other biomarkers are suggested in the guidelines to confirm immune functionality, including the phagocytosis of macrophages, the proliferation of spleen cells, the measurement of the number of plaque-forming cells (PFCs), the expression of T lymphocyte transcription factor, and antigen recognition abilities through the expression of major histocompatibility (MHC) class and toll-like receptors (TLRs). In addition, significant differences were observed in the active ingredient content between the extract and the fermentation result. We hypothesize that components with increased levels in the fermented product contribute to its enhanced immunity-boosting activity. To further investigate this, additional studies are needed using RAW264.7 macrophages and BV2 microglia to assess the activity of these increased components. Moreover, in vivo experiments involving mixtures of ASE with probiotic strains or the fermented product are required.

## 5. Conclusions

In summary, the current study demonstrates that the fermented product of *Acanthopanax sessiliflorus* fruit with *Lactobacillus helveticus* exhibits immune enhancement activity in RAW264.7 macrophages and BV2 microglial cells. Treatment with ASE-LHF increased the production levels of NO and the expression of iNOS and COX-2 proteins. In addition, this fermented product augmented the production of pro-inflammatory cytokines, including IL-6 and TNF-α. The enhancing activity of ASE-LHF was regulated by the activation of the NF-κB and ERK MAPK signaling pathways. In addition, comparison of the metabolomic differences between ASE and its fermented products revealed that phenolic compounds increased after fermentation. This could explain why fermented ASE showed increased activity in our study. Therefore, this investigation suggests that ASE-LHF could be a potential ingredient in health functional foods to improve immunity.

## Figures and Tables

**Table 1:** Relative percentages of secondary metabolites in ASE and fermented ASE (%).

No.	Compound Name	Observed RT (min)	Formula	Neutral Mass (Da)	Observed *m*/*z*	Adducts	Average of Relative Percentage (%)	
							ASE	Fermented ASE
1	22α-hydrochiisanoside	4.32	C_48_H_74_O_20_	971.1	1015.41	+COOH	0.61	0.14
2	Chlorogenic acid	5.13	C_16_H_18_O_9_	354.31	353.16	-H	0.44	0.05
3	Chiisanoside	5.48	C_48_H_74_O_19_	955.1	999.42	+COOH	1.19	N.D
4	3-O-β-D-Glucuronopyranosyl-gypsogenin-28-O-β-D-glucopyranoside	5.78	C_42_H_64_O_15_	808.9	853.38	+COOH	0.38	0.03
5	Acanthosessilioside H	6.27	C_48_H_76_O_19_	957.1	955.44	-H	1.6	-
6	Gracilistylacid B	7.9	C_31_H_48_O_5_	500.7	499.31	-H	6.01	1.96
7	Acanthopanaxoside E	8.38	C_42_H_66_O_15_	811	809.40	-H	5.08	1.8
8	hederagenin-3-O-glucuronopyraoside	9.39	C_36_H_56_O_10_	648.8	647.32	-H	6.14	1.34
9	Silphioside G	10.07	C_42_H_66_O_14_	795	793.41	-H	7.12	1.06
10	28-Hydroxy-28-oxoolean-12-en-3-yl-3-O-xylopyranosyl-glucopyranosiduronic acid	10.65	C_41_H_64_O_13_	764.9	763.40	-H	5.8	0.73
11	Laciniatoside V	11.81	C_27_H_38_O_14_	586.6	631.38	+COOH	6.25	0.97
12	Chiisanogenin	12.08	C_30_H_44_O_5_	484.31	483.32	-H	3.94	3.67
13	[R-(E)]-1-[8-(β-D-Glucopyranosyloxy)-2,6-dimethyl-2-octenoate] β-D-glucopyranose	1.25	C_22_H_38_O_13_	510.5	555.28	+COOH	0.07	2.13
14	Ficusequilignan A	1.65	C_31_H_36_O_11_	584.6	584.30	-H	0.09	3.05
15	4-(1,3-Dihydroxy-2-{4-[(1E)-3-hydroxy-1-propen-1-yl]-2,6-dimethoxyphenoxy}propyl)-2-methoxyphenyl-β-D-allopyranoside	2.15	C_27_H_36_O_13_	568.6	612.33	+COOH	0.23	2.18
16	3-O-[(α-L-Rhamnopyranosyl)(1→2)]-[β-D-glucuronopyranosyl-6-O-methyl ester]-olean-12-ene-28-olic acid	3.52	C_43_H_68_O_12_	776.9	819.39	+COOH	N.D	1.12
17	Feruloylquinic acid-hexoside	3.7	C_23_H_30_O_14_	530.5	528.28	-H	0.02	0.32
18	a-terpineol 8-O-β-D-glucopyranoside	4.68	C_23_H_33_O_10_	468.5	512.27	+COOH	0.03	0.4
19	Caffeoylquinic acid hexoside	5.54	C_22_H_28_O_14_	516.4	515.29	-H	0.75	7.8
20	16α-Hydroxy-17-methylbutyryloxy-ent-kaur-19-oic acid	6.69	C_25_H_39_O_5_	419.6	464.31	+COOH	0.24	6.83
21	5-O-Caffeoyl-3-O-pcoumaroylquinic acid	7.03	C_25_H_24_O_11_	500.4	498.29	-H	0.08	1.64
22	3-O-Caffeoyl-4-O-pcoumaroylquinic acid	7.37	C_25_H_24_O_11_	500.4	498.29	-H	0.46	5.28
23	1-O-p-hydroxybenzoyl-β-D-apiofuranosyl-(1→6)-β-D-glucopyranoside	9.11	C_18_H_24_O_13_	448.4	448.31	-H	0.02	3.37

N.D = Not detected.

## Data Availability

All of the data is contained within the article.
